# Clinical Tests of Hearing—III

**Published:** 1909-10-30

**Authors:** 


### Otology.

### CLINICAL TESTS OF HEARING—III.

### DETECTION OF MALINGERING.

In this country, where there is no compulsory military service, the simulation of deafness is attempted principally after injury, in the hope of obtaining damages, and has become increasingly common since the passing of the Workmen's Compensation Acts. For the purposes of testing and detection, the forms of deafness imitated may be divided into three classes, namely, (1) partial deafness, (2) complete deafness of both ears, and (3) complete deafness of one ear. As to the ways in which genuine deafness may be produced by trauma, it should be remembered that complete, or nearly complete, deafness can only occur when the perceptive apparatus has been considerably damaged. This may result from the sudden loud noise of an explosion, occasionally from concussion of the labyrinth associated with cerebral concussion, but far more commonly from fracture of the base of the skull passing through the petrous bone. The latter will necessarily be associated with loss of consciousness, rupture of the drum, and discharge of blood from the ear; but the last sign may be overlooked at the time, especially if there is also bleeding from a wound on the scalp or auricle, and the ruptured drum may heal rapidly without leaving an obvious scar. A simple rupture of the drum does not cause extreme deafness even at the time, and if it heals quickly produces no permanent impairment of hearing; but, if infection occurs, some degree of deafness will be associated with the resulting suppurative otitis. Therefore it may be said that an alleged severe deafness after injury should be looked on with some suspicion if there is no history of loss of consciousness at the time of injury. Traumatic lesions of the internal ear are usually also accompanied by giddiness and often by tinnitus. In any case, the deafness imitated will be of the type of nerve-deafness, for the subject will not show increased bone-conduction on testing nor hear the tuning-fork on the vertex louder in the "deaf" ear.

#### I. Partial Deafness.

This is rarely imitated, either as unilateral or bilateral deafness, for it is too easy to detect. The subject should be seated in the middle of the room with the eyes blindfolded, and tested repeatedly with acoumeter and tuning-forks. It is impossible under these conditions to give consistent replies; unless the deafness is genuine, the subject will sometimes hear the acoumeter at five feet, and sometimes not at all at three feet. If the case is likely to come into court, two observers should always be present and all results written down at once. Attention may also be paid to the hearing at the two ends of the scale. As there is diminished bone-conduction, and therefore presumably nerve-deafness, the hearing should be proportionately lessened for the higher notes; but the malingerer is unlikely to know this, and will soon go wrong.

#### II. Complete Bilateral Deafness.

This is the variety most difficult of detection by formal tests. Unless the injury suffered has been of the nature of an explosion, in which case the loudness of the sound may have damaged the labyrinth, the trauma required to destroy both internal ears must have been very great, and a history of extremely severe head-injury should be obtainable. One formal test is sometimes of value; when a large C° or C′ fork is made to sound in the ear, either by air or bone conduction, the pupils are frequently found to react, a temporary contraction being followed by dilatation. This is a reflex effect which cannot occur unless the sound reaches the auditory nerve, and, while its absence is of no importance, the presence of the reaction is a sure sign of hearing. For the rest, while formal tests are of little use, few malingerers can keep up the deception consistently if under careful observation for several days. It may be sufficient to tell the subject that he has passed the tests and may go. The effect of a sharp sound behind the patient's back may be observed, or the attempt may be made to wake him from sleep by a sudden noise; but the noise must not be too explosive, for the shock or vibration of such a sound may be perceived by a genuinely deaf person.

#### III. Complete Unilateral Deafness.

According to the writer's experience, complete or nearly complete deafness of one ear is the form most often simulated. It can generally be detected by careful examination. The patient should first be blindfolded and seated in the middle of the room with the "deaf" ear turned towards the examiner and tested by spoken words. He will frequently "fail" to hear them, forgetting that he can hear with the other ear. If he says he hears only with the other ear this can be stopped with a stiff piece of rubber tubing, and, if he now fails to hear, he is obviously deceiving; if he still hears only in the "good" ear he is probably genuinely deaf, for, if he really hears with the "deaf" ear he will find it almost impossible to tell whether the "good" ear is properly stopped or not. If a loud vibrating fork be placed on the "deaf" mastoid and he claims to hear nothing he is probably malingering, for he should hear it plainly by bone-conduction to the other ear. A good test is to block secretly one tube of a binaural stethoscope with moist cotton-wool, to place the ear-pieces in the subject's ears and to whisper into the chest-piece, telling him to repeat the words he hears. If the blocked tube be placed in the "good" ear he is malingering if he hears. If he hear nothing the tubes are reversed and he should then hear, as the open tube is in the "good" ear, but the malingerer is frequently over-cautious and fails to hear in either position. In this way, if the tubes are changed without arousing the subject's suspicions, and especially if the eyes are bandaged, simulated unilateral deafness can nearly always be detected.

